# Auditory function in symptomatic patients recovered from SARS-CoV-2 and unexposed patients: An analytical cross-sectional study

**DOI:** 10.1016/j.joto.2023.05.004

**Published:** 2023-05-19

**Authors:** Katherin Andrea Borda Pedraza, Sergio Mauricio Moreno Lopez, Javier Amaya-Nieto, Liliana Akli Serpa, Ginna Paola Saavedra Martínez, Mauricio Ernesto Quinche Pardo, Alberto Peña Valenzuela

**Affiliations:** a Otorhinolaryngology Department, Universidad Nacional Colombia, Colombia; b Epidemiology Department, Universidad de Los Andes, Colombia; c Health Systems and Services Research Group, Universidad Nacional de Colombia, Bogota, Colombia; d Audiology Department, Universidad Nacional de Colombia, Colombia; e Health and Social Protection Ministry, Colombia; f Universidad Nacional Colombia, Colombia; g Otorhinolaryngology Department, Universidad Nacional de Colombia, Colombia

**Keywords:** SARS-CoV-2, Cochlear diseases, Pure tone audiometry, Otoacoustic emissions, Hearing loss, Otological symptoms

## Abstract

**Objective:**

To describe audiological symptoms, audiometric profile, and distortion product otoacoustic emission in symptomatic patients recovering from SARS-CoV-2 infection (positive RT-PCR test) and asymptomatic patients (negative RT-PCR test).

**Methods:**

An analytical cross-sectional study was conducted using data obtained from clinical charts, physical examination, audiometry, and distortion product otoacoustic emission on 40 patients [case patients (CP)] recovering from SARS-CoV-2 infection diagnosed by a positive RT-PCR test and 22 asymptomatic participants with a negative RT-PCR test [non-case (NC)].

**Results:**

Sixty-two patients (mean age: 31.1 and 28.2 years in the CP and NC groups, respectively) were included. All participants were young without significant comorbidities, risk factors for hearing loss or otological history. Vertigo (5%), tinnitus (17.5%) and aural fullness/hearing loss (35%) were found in the CP group. A statistically significant difference was found in specific frequencies (1000, 4000, and 8000 Hz) and pure tone average (low and high conversational frequencies with increased threshold in the PC group compared with the NC group), which was not found in distortion product otoacoustic emission.

**Conclusion:**

Audiovestibular symptoms are frequent in symptomatic patients recovering from SARS-CoV-2 infection. SARS-CoV-2 infection was consistently associated with an increased audiometric hearing threshold at specific frequencies and low tone average.

## 1 Introduction

The first case of SARS-CoV-2 infection was reported in Wuhan, China, in December 2019. This infection was initially characterized by atypical respiratory symptoms and pneumonia, with high transmissibility and associated mortality, all of which generated a worldwide health crisis, triggering changes in the dynamics of life, health, and the global economy (Mallah et al., 2021). Before vaccination, 87% of those infected were aged between 30 and 79 years, with 81% presenting as asymptomatic or with mild respiratory symptoms, 15% requiring hospitalization, and 3%–4% requiring invasive mechanical ventilation (IVM) and an intensive care unit (ICU). The estimated mortality ranged from 0.39% to 4% (Wu and McGoogan, 2020), with the associated risk factors being an age of >60 years (OR: 8.5, 95% CI: 1.6–44.8), smoking history (OR: 14.2, 95% CI: 1.5–25) and respiratory failure and initial presentation (OR: 8.7, 95% CI: 1.9–40) (Liu et al., 2020).

Transmission occurs through droplets and aerosols, with an incubation period of 4–5 days before the onset of respiratory symptoms. Other symptoms such as headache, dizziness, generalized weakness, vomiting, and diarrhea have been documented (Tay et al., 2020). SARS-CoV-2 has exhibited tropism to the nervous system and has been associated with encephalopathy, acute myelitis, stroke, Guillain–Barré syndrome, and encephalitis (Kilic et al., 2020). Other symptoms include anosmia, dysgeusia, involvement of the audiovestibular system, tinnitus, and vertigo (Lechien et al., 2020). Furthermore, alterations in audiometry and otoemissions in acute frequencies have been found even in patients without otological symptoms (Dharmarajan et al., 2021).

Recently, Jeong et al. demonstrated that the human inner ear tissue expresses the ACE-2 receptor, a transmembrane serine protease 2 (TMPRSS2), and FURIN cofactors, which are required for the virus to enter the hair cells and Schwann cells of the audiovestibular system. They also reported findings from 10 patients with confirmed SARS-CoV-2 infection, in whom audiovestibular dysfunction was identified within 3 weeks of diagnosis. Among the symptoms reported in this group of patients, sudden hypoacusis was identified and treated with corticosteroids, resulting in an improvement after this management (Jeong et al., 2021).

Considering the above-described pathophysiological mechanisms, some authors have investigated the relationship between SARS-CoV-2 infection and alterations in the audiological profile at the clinical level. For instance, Mustafa et al. (Mustafa, 2020) found a decrease in wave amplitude in transient otoacoustic emissions and an increase in high-frequency pure tone thresholds on audiometry in patients with confirmed SARS-CoV-2 infection. Furthermore, Burak et al. (Öztürk et al., 2022) and Aydin et al. (2022) confirmed these findings and suggested vestibulocochlear damage following SARS-CoV-2 infection.

As mentioned earlier, there is suggestive evidence that SARS-CoV-2 infection could affect the nervous tissue of the auditory system and thus diminish the hearing ability of patients with the infection. This study was conducted to evaluate the hearing function of patients with and without a recent history of SARS-Cov-2 infection diagnosed based on RT-PCR results, aged 20–45 years, with no previous comorbidities.

## 2 Materials and methods

### 2.1 Study design

This was an observational, analytical, cross-sectional study describing the audiological profile composed of audiometry and otoacoustic emissions of a group of case patients (CP) consisting of subjects aged 20–45 years, without comorbidities, recovered from symptomatic SARS-CoV-2 infection diagnosed by an RT-PCR test. The non-case (NC) group included asymptomatic patients with a negative RT-PCR result for SARS-CoV-2. This study was conducted between August 2020 and May 2021 at the Hospital Universitario Nacional (HUN) de Colombia in Bogota, Colombia, and was approved by the institutional ethics committee (approval code CEI-2020-07-10). Moreover, the study followed the ethical guidelines established in the Declaration of Helsinki.

### 2.2 Study population

Patients and healthy subjects attending the HUN de Colombia during the pandemic between August 2020 and May 2021 were recruited. The case group was formed from patients who had recovered from symptomatic SARS-CoV-2 infection, as demonstrated by a positive RT-PCR test. At the time of evaluation, these patients were between 3 weeks and 8 months post-infection and did not present symptoms of acute SARS-CoV-2 infection or long COVID symptoms. The non-case group was composed of healthy participants specifically recruited for the study, mainly college students, professors, and office workers from the National University of Colombia. These individuals were recruited through social media and official university email. Preselection of non-cases was done via a Google form to rule out exclusion criteria and SARS-CoV-2 symptoms. All participants in non-case group were tested for SARS-CoV-2 using RT-PCR and were found to be negative.

Both groups met the following inclusion criteria: i) age 20–45 years; ii) absence of significant comorbidities (uncontrolled diabetes mellitus, chronic renal failure, cardiovascular disease, cancer); iii) no documented previous hearing loss and iv) absence of risk factors for hearing loss (occupational noise exposure, exposure to ototoxicants, repeated otitis media, autoimmune diseases, demyelinating diseases or meningitis).

Moreover, both groups met the following exclusion criteria: i) presenting chronic ear pathology identified by questioning or otoscopy (secretion and tympanic perforation) and ii) presenting a history of hearing pathology (sudden sensorineural hearing loss, chronic suppurative otitis media, congenital hearing loss, ear malformations or ear surgeries).

### 2.3 Methodology

We conducted a non-probabilistic, consecutive sampling, recruiting all subjects who met the eligibility criteria until the sample size was completed. A frequency of 8 Hz was used for the calculation, based on the study by Mustafa (2020), and because it is the frequency with the highest variability and thus would maximize the sample size. A difference of 2 dB, a standard deviation of 2 dB for CP and 2.5 dB for NC, with a power of 85%, a significance level of 5% and a 1:1 ratio were established for the following sample size formula:

$$n_{1}=\tau n_{2}\;y\;n_{2}=\frac{{\left(z_{\frac{\alpha }{2}}+z_{\beta }\right)}^{2}s^{2}\left(1+\frac{1}{\tau }\right)}{\varepsilon^{2}}$$

where

$$\tau =\frac{n_{1}}{n_{2}}=1$$

$$s^{2}=\frac{{\left(n_{1}-1\right)s}_{1}^{2}+\left(n_{2}-1\right)s_{2}^{2}}{n_{1}+n_{2}-2}$$

$$\varepsilon =\mu_{1}-\mu_{2}$$

According to the formula, a minimum of 80 ears would be needed, and with a loss adjustment of 10%, the total sample size would be 90 ears (45 for each group).

All study participants underwent a complete medical history-taking, including the characterization of respiratory and otorhinolaryngologic symptoms associated with the audiovestibular system and if presented during the infection. The above-described inclusion and exclusion criteria were applied, and otoscopy was performed to rule out associated pathology of the external and middle visible ear. After ruling out this issue, the informed consent form was signed by the participant, and audiological tests were performed.

Impedance testing was performed to rule out middle ear pathology, classifying the patients into curves type A, Ad, As, B, and C, the last two being considered to be pathological. Subsequently, participants underwent conventional pure tone audiometry to obtain thresholds in decibels (dB) at frequencies between 250 and 8000 Hz for individual analysis and by an average of conventional pure tone PTA as follows: low-tone PTA (500, 1000, 2000 and 3000 Hz) and high-tone PTA (4000, 6000 and 8000 Hz). Otoacoustic emissions were also analyzed using extended range distortion product between 700 and 8000 Hz for the analysis of their presence or absence and their corresponding amplitude. For the evaluation of the signal-to-noise ratio (S/R, i.e. the difference between the noise floor and the otoemission amplitude value), a result of <7 dB was considered as negative otoemission, that between 7 and 10 dB was considered as otoemission presenting with decreased amplitude and a result >10 dB was considered as otoemission presenting with normal amplitude, according to IEC 60601–1 and IEC 60645-6 standards for the Eclipse DPOAE20 equipment of Interacustics A/S at the HUN.

### 2.4 Statistical analysis

Qualitative variables were described using absolute and relative frequencies, and quantitative variables were described using measures of central tendency and dispersion, according to their nature.

Differences between each of the audiometry frequencies within groups were calculated using a Mann–Whitney test, which was performed for PTA values at low frequencies (500, 1000, 2000 and 3000 Hz) and high frequencies (4000, 6000, 8000 Hz). Similarly, the otoemission values were compared between the study groups for frequencies from 1 to 8 kHz using a Wilcoxon signed-rank test. The significance level established a priori was 5%, and the software Stata 17 MP was used for statistical analyses.

## 3 Results

A total of 62 participants corresponding to 124 ears were recruited and divided into two groups; the CP group consisted of 40 participants corresponding to 80 ears (64.5%), and the NC group consisted of 22 participants corresponding to 44 ears (35.5%).

Participants in the CP group had a mean age of 31.05 years (SD = 8.19) and a median age of 30.00 years (RIQ = 24.00–38.00), and men accounted for 45.00% (n = 18). In this group, two patients had controlled hypothyroidism, and four patients received azithromycin management for SARS-CoV-2 infection on a short 3-day schedule. Participants in the NC group had a mean age of 28.27 years (SD = 6.26) and a median age of 28.27 years (RIQ = 6.26), and men accounted for 36.36% (n = 8).

A characterization of symptoms was performed in the CP group, which revealed headache as the most frequent symptom (67.5%; n = 27), followed by general malaise (65.0%; n = 26), cough (35.0%; n = 14) and fever (32.5%; n = 13). Among the symptoms associated with the otorhinolaryngologic system, the most frequent was an alteration of taste (45%; n = 18) and smell (50%; n = 20). Regarding the audiovestibular system, vertigo was found in 5% (n = 2), tinnitus was found in 17.5% (n = 7) and sensation of fullness/hearing loss was found in 35% (n = 14) of participants. The median number of days of illness was 14.00 (RIQ = 13.00–15.00). Infection severity was identified in our population according to where treatment was received (outpatient, hospitalization or ICU), and the proportions are presented in Table 1.

**Table 1:** Symptomatologic and severity characteristics in the group of case patients (CP).

Variable	Case patients
	N = 40
Symptoms – n (%)	
Cough	14 (35.00)
Fever	13 (32.50)
Odynophagia	6 (15.00)
General malaise	26 (65.00)
Headache	27 (67.50)
Dyspnea	6 (15.00)
Taste alterations	18 (45.00)
Gastrointestinal symptoms	8 (20.00)
Smell alterations	20 (50.00)
Sinus symptoms	9 (22.50)
Tinnitus	7 (17.50)
Vertigo	2 (5.00)
Sensation of aural fullness/hearing loss	14 (35.00)
Place of treatment (%)	
Home	35 (87.50)
Hospitalization floor	4 (10.00)
UCI	1 (2.50)
Median number of days in isolation or hospitalization (IQR)	14.00 (13.00–15.00)

**Fig. 1:**
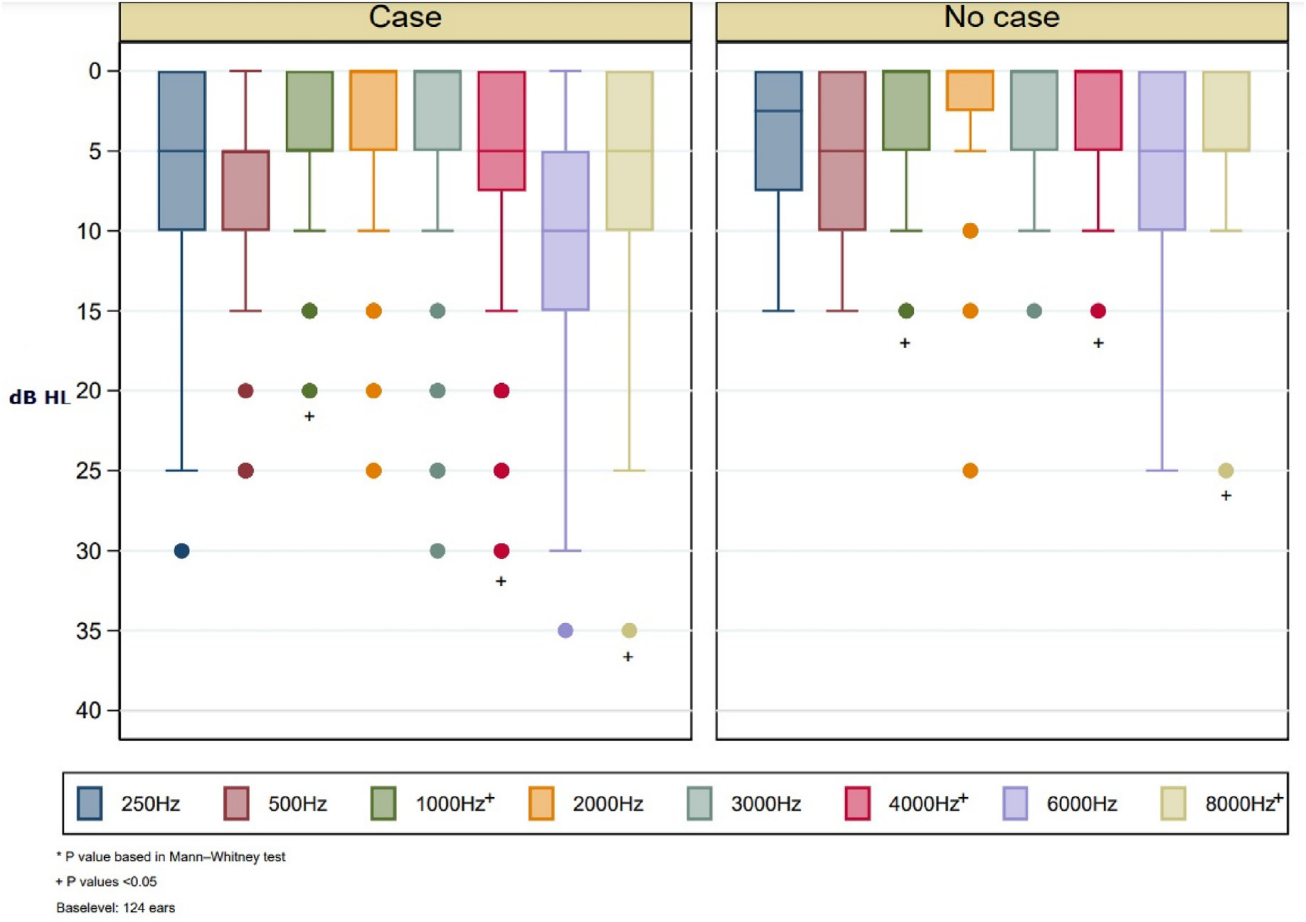
Box plot thresholds for specific frequency comparing between CP and NC groups.

**Fig. 2:**
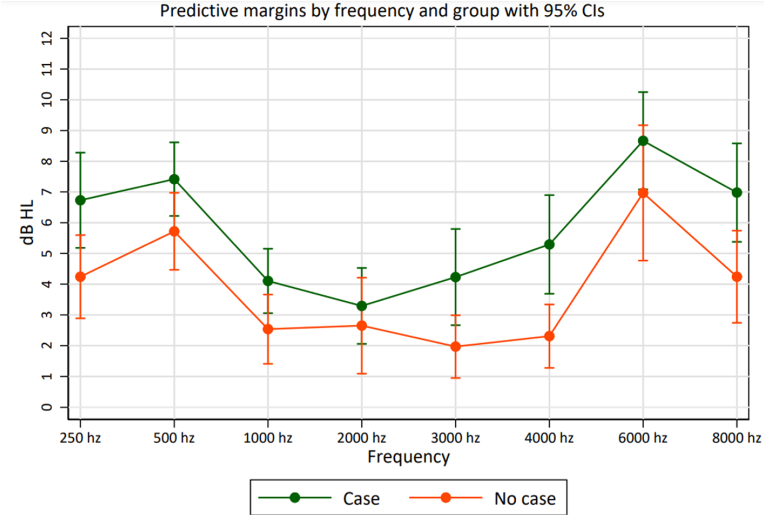
Graphic of cluster regression analysis for frequencies in case (CP) and non-case (NC) groups.

The median threshold values in decibels (dB) at each frequency (250, 1000, 2000, 3000, 4000, 6000, and 8000 Hz) in the standard audiometry were reported for the CP and NC groups. There was a statistically significant difference in the values corresponding to the frequencies of 1000, 4000, and 8000 Hz (p < 0.05), with higher thresholds for the CP group than for the NC group (Table 2 and Fig. 1). Additionally, a cluster regression analysis was performed using a GEE (Generalized Estimating Equation) model with robust errors and an unstructured correlation matrix. The model was adjusted for sex, age, and laterality to establish possible differences between the comparison groups. A mean difference of 2.98 dB was found between cases and non-cases for frequencies of 4 KHz (95% CI: [−4.89; -1.08], p < 0.05), as shown in Table 3. This was the largest difference observed. The results are consistent with those previously reported (Fig. 2).

**Table 2:** Audiometry results for CP and NC groups by specific frequency and in tone average of conversational frequency: low (500, 1000, 2000 and 3000 Hz) and high (4000, 6000, 8000 Hz).

Frequency (a)	CP group	NC group	Total	p-value (b)
	N = 80	N = 44	N = 124	
Air conduction values (dB HL)				
250	5.00 (0.00–10.00)	2.50 (0.00–7.50)	5.00 (0.00–10.00)	0.060
500	5.00 (5.00–10.00)	5.00 (0.00–10.00)	5.00 (5.00–10.00)	0.15
1000	5.00 (0.00–5.00)	0.00 (0.00–5.00)	0.00 (0.00–5.00)	0.020∗
2000	0.00 (0.00–5.00)	0.00 (0.00–2.50)	0.00 (0.00–5.00)	0.22
3000	0.00 (0.00–5.00)	0.00 (0.00–5.00)	0.00 (0.00–5.00)	0.10
4000	5.00 (0.00–7.50)	0.00 (0.00–5.00)	0.00 (0.00–5.00)	0.020∗
6000	10.00 (5.00–15.00)	5.00 (0.00–10.00)	5.00 (0.00–15.00)	0.11
8000	5.00 (0.00–10.00)	5.00 (0.00–5.00)	5.00 (0.00–10.00)	0.024∗
Low PTA	2.50 (1.25–6.25)	2.50 (1.25–4.38)	2.50 (1.25–5.00)	0.035∗
High PTA	6.67 (1.67–10.00)	3.33 (0.83–6.67)	5.00 (1.67–8.33)	0.010∗

∗ 95% confidence interval with statistical significance.

a Data are presented as median (IQR) for continuous measures.

b p values based on the Wilcoxon rank-sum test.

**Table 3:** Cluster regression analysis for frequencies in case (CP) and non-case (NC) groups.

Frecuency	Delta-method					
	dy/dx	std. err.	z	P>|z|	[95% conf. interval]	
	base outcome					
250 Hz	−2,49	1,04	−2,38	0,02	−4,53	−0,44
500 Hz	−1,70	0,88	−1,92	0,05	−3,43	0,03
1000 Hz	−1,57	0,78	−2,01	0,04	−3,10	−0,04
2000 Hz	−0,64	1,00	−0,64	0,52	−2,61	1,33
3000 Hz	−2,26	0,95	−2,37	0,02	−4,13	−0,39
4000 Hz	−2,98	0,97	−3,07	0,00	−4,89	1,08
6000 Hz	−1,70	1,38	−1,23	0,22	−4,40	1,00
8000 Hz	−2,74	1,13	−2,43	0,02	−4,95	−0,53

Note: dy/dx for factor levels is the discrete change from the base level.

**Fig. 3:**
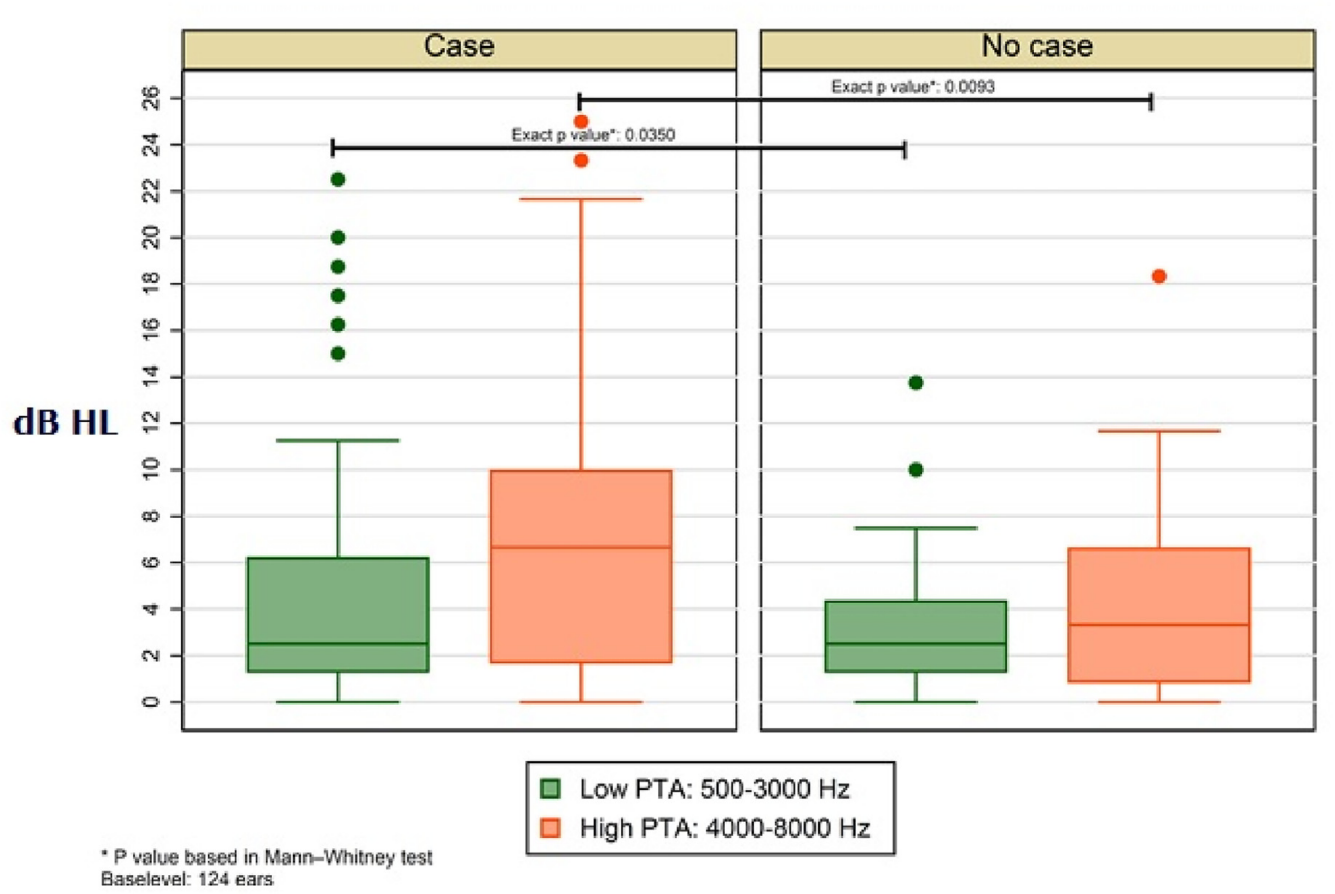
Box plot with average of PTA conversational tones and average of high PTA high frequencies in case (CP) and non-case (NC) groups.

The analysis of the average of tones for standard frequencies showed in the low PTA (500, 1000, 2000, 3000 Hz), when comparing groups, a difference (p < 0.05) in favor of higher thresholds in the CP group. Moreover, when analyzing high PTA frequencies (4000, 6000, and 8000 Hz), a statistically significant difference of 3.33 dB (p < 0.05) was observed for the CP group compared with the NC group (Fig. 3).

**Fig. 4:**
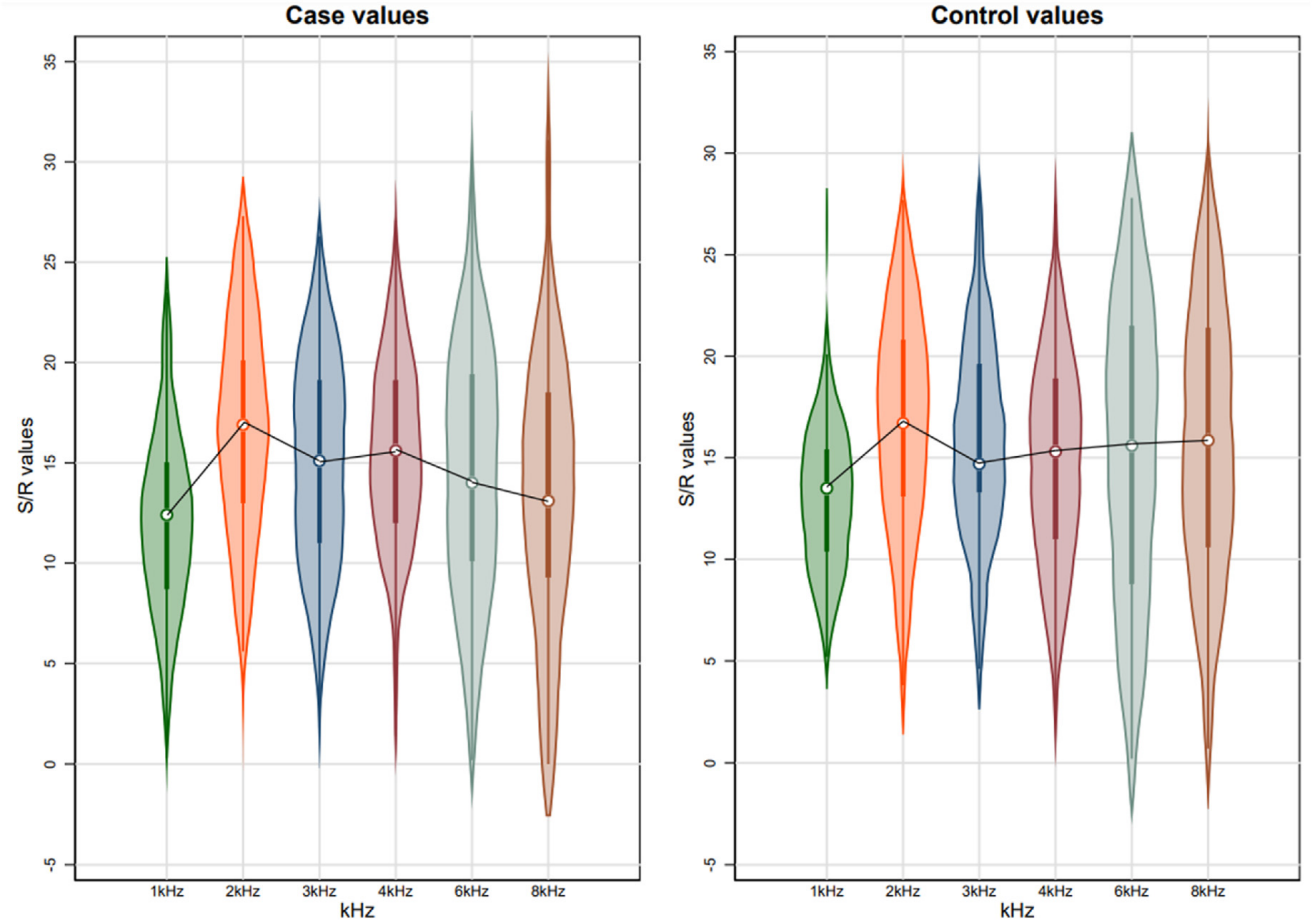
Violin Plot: Comparison of otoacoustic emissions by frequency between case and non-case groups.

The distortion product of the otoacoustic emission in CP and NC groups was analyzed using the ratio between the amplitudes and S/R ratio at frequencies of 1000, 2000, 3000, 4000, 6000, and 8000 Hz. No difference was found in any of the frequencies analyzed between the groups (Table 4 and Fig. 4).

**Table 4:** Frequency-specific distortion product otoemission results for both groups. Frequency (a) Group CP Group NC Total p-value (b).

Frequency ^(^a^)^	Group CP	Group NC	Total	p-value ^(^b^)^
	N = 80	N = 44	N = 124	
Otoemission values (S/R)				
1000	12.40 (8.70–15.00)	13.50 (10.40–15.40)	12.50 (9.45–15.05)	0.17
2000	16.90 (13.00–20.10)	16.70 (13.10–20.80)	16.90 (13.00–20.20)	0.99
3000	15.10 (11.00–19.10)	14.70 (13.30–19.60)	15.00 (11.50–19.40)	0.46
4000	15.60 (12.00–19.10)	15.30 (11.00–18.90)	15.30 (11.50–18.90)	0.56
6000	14.00 (10.10–19.40)	15.60 (8.80–21.50)	14.95 (9.40–19.80)	0.70
8000	13.10 (9.30–18.50)	15.85 (10.60–21.40)	13.50 (9.60–19.60)	0.17

a Data are presented as median (IQR) for continuous measures.

b p values based on the Wilcoxon rank-sum test.

## 4 Discussion

It is well known that hearing loss is related to virus infection. Clinical manifestations can range from various symptoms, including congenital or acquired, unilateral or bilateral, sudden or progressive, and mild to profound hearing loss (Cohen et al., 2014; Chandrasekhar et al., 2019). In the context of the SARS-CoV-2 pandemic, it has been found that this virus can affect neural cells of the audiovestibular system, manifesting itself clinically with vertiginous symptoms and hearing loss (Jeong et al., 2021). In addition to the classic respiratory symptoms, audiovestibular symptoms have been reported after an acute SARS-CoV-2 infection. A meta-analysis conducted by Zahra et al. (Jafari et al., 2022) in 2021 reported that the rates of occurrence of hearing loss, tinnitus, and vertigo in cases with confirmed SARS-CoV-2 were 3.1% (95% CI: 0.01–0.09), 4.5% (95% CI: 0.012–0.153) and 12.2% (95% CI: 0.070–0.204), respectively. In our study, the frequencies of these symptoms were 35%, 17.5% and 5%, respectively.

Multiple reports have been published associating sudden hearing loss with SARS-CoV-2 infection (Kilic et al., 2020; Degen et al., 2020). Recently, Meng et al. (2022) published a systematic review of case reports, finding 23 reports of sudden hearing loss associated with SARS-CoV-2 infection to date. The mean age of these patients was 43.1 years, and the presentation was unilateral in 60.8% and bilateral in 39.2% of cases. Hearing loss was identified as the only symptom of infection in 17.4% of cases. The majority (82.6%) of these patients had a clinical presentation accompanied by one or more symptoms such as tinnitus (60.9%), vertigo (13.0%), dizziness (8.7%), ear pain (4.0%) and peripheral facial nerve palsy (4.0%). In our study sample, there were no patients who met the criteria for sudden hypoacusia. However, it must be emphasized that infection can manifest with this clinical presentation, as evidenced by the current literature.

To exactly define hearing loss, different studies have aimed to compare pure tone audiometry, transient otoemission and distortion product audiometry in patients with SARS-CoV-2 infection. The study by Osman et al. (Durgut et al., 2022) on 20 patients compared the audiometry results before and after mild SARS-CoV-2 infection and reported no statistically significant differences in audiometric thresholds for all the evaluated frequencies. Another prospective study by Bhattano et al. (Bhatta et al., 2021) included 331 patients with SARS-CoV-2 infection and 80 control subjects and reported no significant differences in audiometric thresholds at any audiometric frequencies. Furthermore, a study by Yıldız (2022) on a population of 240 patients divided into three groups of 80 patients (controls, SARS-CoV-2-infected patients without pneumonia, and SARS-CoV-2-infected patients with pneumonia) found no significant differences in pure tone audiometry or transient otoemissions between these groups.

Although the low or high PTA in the CP group did not exceed 15 dB (which would define clinically mild associated hearing loss), there was a statistically significant difference of 3.34 dB (p = 0.010) in the values of high PTA between the CP and NC groups using a Wilcoxon signed-rank test. Although these findings are not clinically significant, they may reflect low variability between groups with minimal differences that could be detected by the statistical test. This suggests a possible incipient process of deterioration, sequelae, or injury to the audiovestibular system associated with the infection. This positive correlation was also found in previous studies where an association between SARS-CoV-2 infection and hearing loss was detected, as reported by Dharmarajan et al. (2021). They used a sample of 100 patients with SARS-CoV-2 infection and identified a 53% prevalence of high-frequency sensorineural hearing loss and 49% of patients with referred otoemissions in both ears. Conversely, Mustafa et al. reported differences (p < 0.05) in the acute frequency thresholds of 4000, 6000, and 8000 Hz and in the amplitude of transient otoemissions (p < 0.001) when comparing asymptomatic patients with SARS-CoV-2 infection with uninfected patients (Mustafa, 2020). In addition, statistically significant differences have been reported in all frequencies evaluated in patients with hearing loss associated with SARS-CoV-2 infection compared with controls (Dusan et al., 2022), as well as in specific acute frequencies (between 4 and 14 kHz), with a decrease in amplitudes of transient otoemissions (at frequencies of 1500, 2000, and 4000 Hz) and in distortion product otoemissions at frequencies of ≥4003 Hz (p < 0.05) (Öztürk et al., 2022). Despite these positive findings regarding amplitude loss in otoemissions, our results in this area do not indicate statistically significant differences. This result can be explained by the different mechanisms of damage observed in SARS-CoV-2 infection, all of which may or may not initially affect the outer hair cells, which were the specific target of study in the acoustic otoemissions. Nevertheless, this finding does not rule out the alteration of other types of cells in the cochlea, audiovestibular nerve, and stromal Schwann cells (Jeong et al., 2021).

There are some limitations in the present study related to the design. As an analytical cross-sectional study, the results are merely associative and cannot be assumed to be causative. Similarly, the selected population was particular as it was composed of only one health insurance group, reducing its extrapolation to populations from other socioeconomic contexts. However, a strength of this study is that it is the first investigation in a middle-income Latin American country exploring the possible audiovestibular involvement in subjects with confirmed SARS-CoV-2 infection. We consider that the present study supports previously published data, which demonstrated the possibility of compromise in the audiovestibular system by SARS-CoV-2 infection. It highlights the importance of clinical history and physical and audiological examinations in patients after recovery from SARS-CoV-2 infection, which can help detect the associated audiovestibular pathology and allow timely follow-up and rehabilitation.

## Ethics approval

The Ethical Committee of the Hospital Universitario Nacional de Colombia (approval code CEI-2020-07-10) approved this study. All patients provided written informed consent.

All authors participated in conducting this study, writing the manuscript, and critically reviewing the intellectual content of the manuscript.

## Funding

This study was supported by the 10.13039/501100002753Universidad Nacional de Colombia and Hospital Universitario Nacional (HUN) de Colombia.

## Declaration of competing interest

The authors report no conflicts of interest. The authors alone are responsible for the content and writing of the paper.

## References

[bib1] Aydin S., Koca C.F., Celik T., Kelles M., Yasar S., Oguzturk S. (2022). The effect of the severity of COVID-19 on the sequelae of the audiovestibular system. Ear Nose Throat J..

[bib2] Bhatta S. (2021). Study of hearing status in COVID-19 patients: a multicentered review. Indian J. Otolaryngol. Head Neck Surg..

[bib3] Chandrasekhar S.S. (2019). Clinical practice guideline: sudden hearing loss (update). Otolaryngol. Head Neck Surg..

[bib4] Cohen B.E., Durstenfeld A., Roehm P.C. (2014). Viral causes of hearing loss: a review for hearing health professionals. Trends Hear.

[bib5] Degen C., Lenarz T., Willenborg K. (2020). Acute profound sensorineural hearing loss after COVID-19 pneumonia. Mayo Clin. Proc..

[bib6] Dharmarajan S. (2021). Hearing loss-A camouflaged manifestation of COVID 19 infection. Indian J. Otolaryngol. Head Neck Surg..

[bib7] Durgut O., Karataş M., Çelik Ç., Dikici O., Solmaz F., Gencay S. (2022). The effects of SARS-CoV-2 on hearing thresholds in COVID-19 patients with non-hospitalized mild disease. Am. J. Otolaryngol..

[bib8] Dusan M., Milan S., Nikola D. (2022). COVID-19 caused hearing loss. Eur. Arch. Oto-Rhino-Laryngol..

[bib9] Jafari Z., Kolb B.E., Mohajerani M.H. (2022). Hearing loss, tinnitus, and dizziness in COVID-19: a systematic review and meta-analysis. Can. J. Neurol. Sci..

[bib10] Jeong M. (2021). Direct SARS-CoV-2 infection of the human inner ear may underlieve COVID-19-associated audiovestibular dysfunction. Community Med..

[bib11] Kilic O., Tayyar M., Cag Y., Tuysuz O., Pektas (2020). Could sudden sensorineural hearing loss be the sole manifestation of COVID-19?. Int. J. Infect. Dis..

[bib12] Lechien J.R. (2020). Olfactory and gustatory dysfunctions as a clinical presentation of mild-to-moderate forms of the coronavirus disease (COVID-19): a multicenter European study. Eur. Arch. Oto-Rhino-Laryngol..

[bib13] Liu W. (2020). Analysis of factors associated with disease outcomes in hospitalized patients with 2019 novel coronavirus disease. Chin. Med. J..

[bib14] Mallah S.I. (2021). COVID-19: breaking down a global health crisis. Ann. Clin. Microbiol. Antimicrob..

[bib15] Meng X., Wang J., Sun J., Zhu K. (2022). COVID-19 and sudden sensorineural hearing loss: a systematic review. Front. Neurol..

[bib16] Mustafa M.W.M. (2020). Audiological profile of asymptomatic Covid-19 PCR-positive cases. Am. J. Otolaryngol..

[bib17] Öztürk B., Kavruk H., Aykul A. (2022). Audiological findings in individuals diagnosed with COVID-19. Am. J. Otolaryngol..

[bib18] Tay M.Z., Poh C.M., Rénia L., MacAry P.A., Ng L.F.P. (2020). The trinity of COVID-19: immunity, inflammation and intervention. Nat. Rev. Immunol..

[bib19] Wu Z., McGoogan J.M. (2020). Characteristics of and important lessons from the coronavirus disease 2019, (COVID-19) outbreak in China: summary of a report of 72314 cases from the Chinese center for disease control and prevention. JAMA, J. Am. Med. Assoc..

[bib20] Yıldız E. (2022). Comparison of pure tone audiometry thresholds and transient evoked otoacoustic emissions (TEOAE) of patients with and without Covid-19 pneumonia. Am. J. Otolaryngol..

